# Diagnostic Challenges of* Cryptococcus neoformans* in an Immunocompetent Individual Masquerading as Chronic Hydrocephalus

**DOI:** 10.1155/2016/7381943

**Published:** 2016-07-20

**Authors:** Kedar R. Mahajan, Amity L. Roberts, Mark T. Curtis, Danielle Fortuna, Robin Dharia, Lori Sheehan

**Affiliations:** ^1^Department of Neurology, Thomas Jefferson University Hospital, Philadelphia, PA 19107, USA; ^2^Department of Pathology, Anatomy and Cell Biology, Thomas Jefferson University Hospital, Philadelphia, PA 19107, USA

## Abstract

*Cryptococcus neoformans* can cause disseminated meningoencephalitis and evade immunosurveillance with expression of a major virulence factor, the polysaccharide capsule. Direct diagnostic assays often rely on the presence of the cryptococcal glucuronoxylomannan capsular antigen (CrAg) or visualization of the capsule. Strain specific phenotypic traits and environmental conditions influence differences in expression that can thereby compromise detection and timely diagnosis. Immunocompetent hosts may manifest clinical signs and symptoms indolently, often expanding the differential and delaying appropriate treatment and diagnosis. We describe a 63-year-old man who presented with a progressive four-year history of ambulatory dysfunction, headache, and communicating hydrocephalus. Serial lumbar punctures (LPs) revealed elevated protein (153–300 mg/dL), hypoglycorrhachia (19–47 mg/dL), lymphocytic pleocytosis (89–95% lymphocyte, WBC 67–303 mg/dL, and RBC 34–108 mg/dL), and normal opening pressure (13–16 cm H_2_O). Two different cerebrospinal fluid (CSF) CrAg assays were negative. A large volume CSF fungal culture grew unencapsulated* C. neoformans*. He was initiated on induction therapy with amphotericin B plus flucytosine and consolidation/maintenance therapy with flucytosine, but he died following discharge due to complications. Elevated levels of CSF Th1 cytokines and decreased IL6 may have affected the virulence and detection of the pathogen.

## 1. Introduction

Cryptococcal infections are primarily due to the following serotypes:* Cryptococcus neoformans* var.* grubii* (serotype A),* C. neoformans* var.* neoformans* (serotype D), AD haploid, and* C. gattii* (formerly* C. neoformans* var.* gattii*) (serotypes B and C) [1, 2]. Global environmental niches for* C. neoformans* var.* grubii/neoformans* are avian (pigeon) guano, soil, and decaying vegetation.* C. gattii* is found in* Eucalyptus camaldulensis*, Douglas fir trees, and surrounding soil [3, 4].* C. neoformans* and* C. gattii* typically infect immunocompromised and immunocompetent individuals, respectively, [5, 6] and often cause significant neurologic morbidity.

*C. neoformans* is a spherical to oval (4–10 *μ*m) narrow-based budding yeast that variably produces a polysaccharide capsule which can trigger complement activation and depletion, impact antibody responsiveness, and inhibit leukocyte migration and macrophage phagocytosis [7]. Capsule components include glucuronoxylomannan (GXM) which interferes with complement mediated phagocytosis [8], galactoxylomannan (GalXM), and mannoprotein [7, 9, 10]. While the main virulence factor is the capsule, others include melanin synthesis, urease and phospholipid secretion, titan cell formation, resistance to host body temperature, and surface phospholipid glucosylceramide (GlcCer) [1, 11]. Capsule formation is induced by environmental and nutritional factors (e.g., iron, carbon dioxide, glucose, amino acids, pH, and temperature [7]). Its size varies with growth conditions, increases in the host during active infection [11], and, even in the same host, variability in capsule thickness and diameter between lung and meningeal tissue has been described [8].

Diagnostics utilize CrAg for antibody based assays (sensitivity and specificity for serum): latex agglutination (LA, 97% sensitivity; 86–100% specificity [12]), enzyme immunoassay (EIA, sensitivity 94%; specificity 96% [13]), and lateral flow assay (LFA, 98.7% specificity; 100% sensitivity; IMMY package insert) 90% [4, 12, 14, 15]. Diagnosis by culture is reliable but takes time. India ink displacement around the capsule allows direct visualization of the yeast cells via light microscopy with high specificity (100%) but limited sensitivity (50%) due to dependence on both cell titer and capsule production [12].

Inhalation of desiccated yeast cells can eventually introduce the organism systemically via a hematogenous route [11], especially in immunocompromised hosts [13]. The organism can be eliminated or remain dormant in immunocompetent hosts.* Cryptococcus* can infect pulmonary, dermatologic, vascular, musculoskeletal, ophthalmologic, genitourinary, cardiac, and endocrine sites with tropism for the central nervous system (CNS) [10, 16].* C. neoformans* transverses the blood-brain-barrier through transcytosis (internalization and transcellular transfer) and phagocytosis-mediated entry via monocytes/macrophages that are emigrating into the CNS [17]. CNS syndromes range from stroke, dementia, meningoencephalitis, abscess, subdural effusion, or spinal cord lesions [18] to recently described focal cranial neuropathies involving the optic chiasm/tracts and cranial nerves VI–VIII [19].

## 2. Case Report

**Figure 1:**
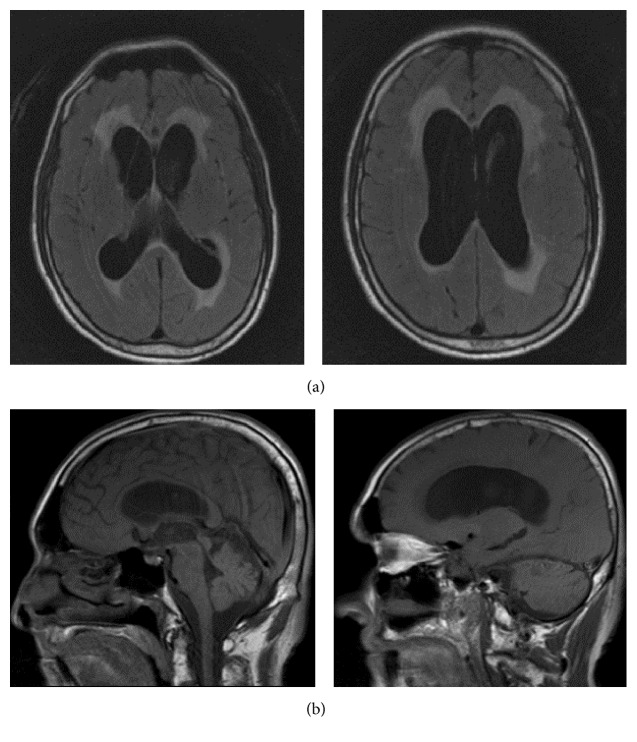
MRI FLAIR axial (a) and sagittal (b) notable for communicating hydrocephalus.

We present the case of a 63-year-old man with progressive gait dysfunction and headache for four years. Computed tomography (CT) head revealed communicating hydrocephalus at an outside institution a year prior to our encounter (Figure 1). He had been offered, but declined, a ventriculoperitoneal shunt (VPS) given his history of frequent falls, ataxia, and dizziness. We additionally learned of his cognitive decline, urinary incontinence, chronic headache, dysarthria, and intermittent walker use. Medical comorbidities included hypertension, hyperlipidemia, and a left cerebellar stroke 3 years earlier with evidence of basal ganglia lacunar infarcts on imaging. He had a 50-pack-year history of tobacco. He was on disability at the time of admission and had most recently lived alone with a pet cockatoo in New Jersey for 6 years. Previously, he had been a truck driver and had resided in Florida for 30 years, during which he had also cleaned wastewater treatment plants. Notably on exam, he was slow to respond and dysarthric and had dysmetria with finger-to-nose and heel-to-shin testing. Gait assessment showed a normal base but severe ataxia, an inability to put feet together, and minimal retropulsion ability.

He had four serial lumbar punctures performed (Table 1), which revealed elevated protein (153–300 mg/dL), hypoglycorrhachia (19–47 mg/dL), lymphocytic pleocytosis (89–95% lymphocytes, WBC 67–303 mg/dL, and RBC 34–108 mg/dL), and normal opening pressure (13–16 cm H_2_O). Gait assessment was variable after large volume lumbar punctures (LPs) with no improvement after the second LP (gait assessment not performed after first LP) and some improvement after the third LP (distance walked improved from 2 to 6 ft in 20 sec and ability to lift legs off the ground more readily) and the fourth LP (ability to get up from the chair more readily and take few steps with less assistance). Empiric treatments while the diagnosis was pending included pulse dose intravenous methylprednisolone, carbidopa/levodopa, and acetazolamide.

**Figure 2:**
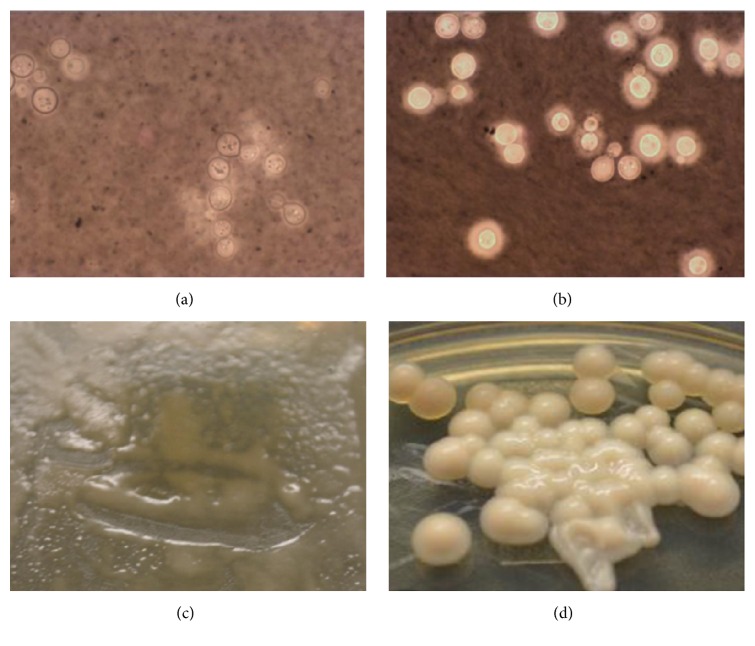
(a and b) India ink of original uncapsulated colony (a) and subbed colony (b) which produced capsule (1000x magnification). (c and d) Original colony on SABs flask; note rough colony phenotype as well as small colony formation (c). Subbed colonies, note abundant capsule production and typical large colony formation (d).

Direct CSF CrAg, both the Remel* Cryptococcus* Antigen LA (Thermo Scientific, Remel, Lenexa, KS) and the IMMY LFA (Immuno-Mycologics, Norman, OK), were performed on 3 of 4 LPs and were negative. Multiple direct antigen tests were utilized due to a high suspicion for cryptococcal meningitis. Fungal cultures were performed on the 1st and 4th collection of which only the 4th (high volume, 31 mL) had a light growth of* C. neoformans* on Sabouraud dextrose agar (SABs) 7 days after inoculation. The 4th collection was tested for prozone effect by both LA and LFA and remained negative. Since the initial colony type did not have typical morphology, a Remel Rapid Yeast ID panel was performed and provided an identification of* C. neoformans*. The rough colonies (Figure 2) were observed with India ink, noting an appropriate cell size but missing the capsule. Identification was confirmed by growth of dark colonies on birdseed agar (melanin production) and production of a positive reaction (pink) on rapid urea media. Additionally, matrix-assisted laser desorption/ionization time of flight mass spectrometry (MALDI-TOF-MS) (Bruker Biotyper MicroFlex, Massachusetts) confirmed the isolate identification as* C. neoformans*. Upon subculture of the original colony to SABs, the colony began reexpressing capsule (Figure 2) as noted by both the colony appearance (white and mucoid) and India ink stain of the subbed colony.

Following the positive culture, he was readmitted for induction with amphotericin B and flucytosine and consolidation/maintenance with flucytosine. He had a prolonged hospital course complicated by an epidural hematoma, deep venous thrombosis, pneumonia, and acute respiratory failure and unfortunately died due to complications.

**Figure 3:**
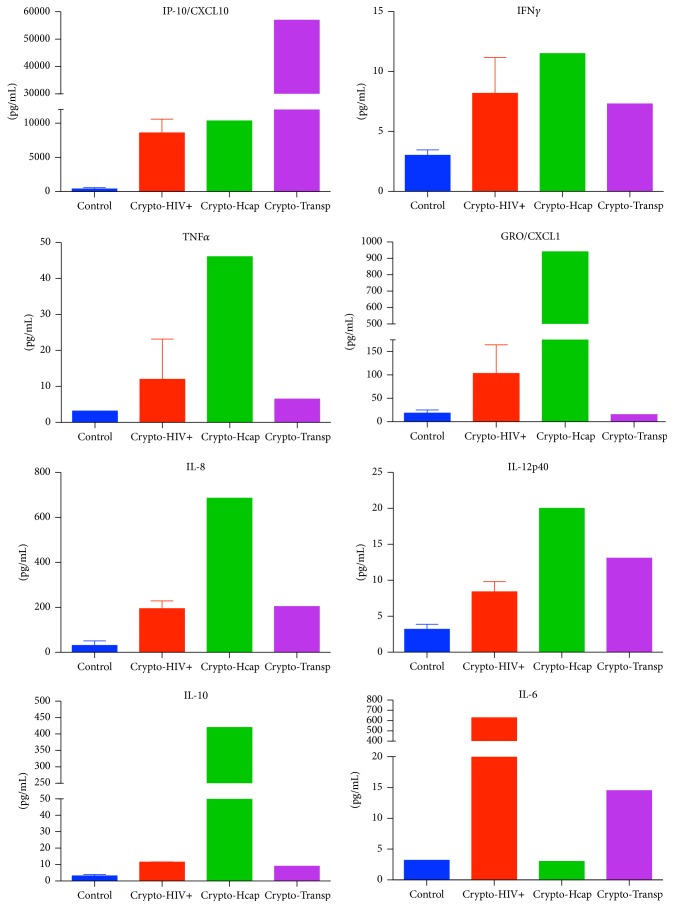
Respective cytokine levels in control (*n* = 6),* Cryptococcus* in HIV-positive (HIV+, *n* = 2),* Cryptococcus* in our patient (hypocapsular (Hcap), *n* = 1), and* Cryptococcus* after cardiac transplant (Transp, *n* = 1).

We sought to determine differences in the patient's immune response compared to individuals either without cryptococcal infection or infected with an immunocompromised state. We retrospectively compared levels of Th1 (IP10/CXCL10, IFN*γ*, TNF*α*, GRO/CXCL1, interleukin (IL)-8, and IL12p40) and Th2 (IL10 and IL6) inflammatory cytokines using a fluorescent bead-based ELISA (Luminex®) in patients with idiopathic intracranial hypertension (six noninfectious controls) and patients with cryptococcal meningoencephalitis (two HIV-positive, our immunocompetent patient, and one after cardiac transplant on immunosuppression). Although we were unable to evaluate statistical significance from inadequate sample size, Figure 3 shows relative higher levels of Th1 cytokines, higher levels of the Th2 cytokine IL10, and a lower level of IL6.

## 3. Discussion

We describe a case of an immunocompetent man with* Cryptococcus neoformans* meningoencephalitis that evaded detection with LA/LFA antigen based assays and one of two fungal cultures. We speculate both pathogen and host immunity that may contribute to poor detection. A low fungal CSF titer and impaired capsule production can contribute to poor detection with antigen based assays, negative staining with India ink, and fungal culture. Additionally, reluctant capsule expression until large volume culture with subculturing suggests that the inoculated strain may have been modulating capsule expression, perhaps in the presence of a competent host immune response. Host cytokine expression, with low IL6 expression, may have altered CrAg shedding. Failure to perform continued* large* volume fungal culturing at* each* lumbar puncture (fungal culture sent only with 1st and 4th LP) may represent missed opportunities.

Capsule-deficient* C. neoformans* strains can produce false-negatives on antigen assays [4]. Host mechanisms such as IL18 production can downregulate GXM, reduce fungal burden, and may contribute to a hypocapsular phenotype [20]. Reports of acapsular* C. neoformans* have been reported with pulmonary disease [21], septic arthritis [22], and meningoencephalitis [23–25].

Garber and Penar describe a young immunocompetent woman presenting with occipital headache, hydrocephalus, elevated intracranial pressure (20–30 cm H_2_O), normal protein and glucose, lymphocytic pleocytosis (WBCs 15, RBCs 5, and 80% lymphs), and CSF culture revealing nonencapsulated* C. neoformans* with a negative antigen test [26]. While our patient did not have elevated intracranial pressure, he did exhibit lymphocytic pleocytosis and hydrocephalus. Del Poeta purports that the capsule is not a prerequisite for infection based on cases of acapsular and hypocapsular strains causing disease and variation in capsular size throughout different phases of infection [27].

Aside from capsule modification, host immune responses may have perturbed detection of the CrAg in the serum and CSF by variable shedding. Boulware et al. describe “low,” “intermediate,” and “high” CrAg shedders normalized to fungal burden in HIV-infected* C. neoformans* patients higher shedding in increased levels of IL6/8 [28]. Lower levels of IL6 in our patient may even have modulated cryptococcal entry into the CNS as IL6 deficiency* in vivo* has been attributed to increased blood-brain-barrier permeability and higher mortality in IL6^−/−^ mice and with neutralizing IL6 antibodies [29]. Because immunocompetent individuals have lower mortality and 10-fold lower serum CrAg titers [30], they likely can harbor* Cryptococcus* for longer periods without manifesting symptoms and delay diagnosis.

Elevated levels of Th1 cytokines, such as IP-10/CXCL10 secretion in response to IFN-*γ*, can be expected in an active infection and are associated with improved survival in* C. neoformans* meningitis [31–33]. Our immunocompetent patient had a more robust Th1 response compared with immunocompromised patients based on observed CSF levels of IFN*γ*, TNF*α*, GRO/CXCL1, IL8, and IL12p40 (Figure 3). Elevated IL10 (human cytokine synthesis inhibitory factor) in our patient, an anti-inflammatory cytokine which promotes a Th2 response, is detrimental to combating* C. neoformans* (Figure 3) and has been associated with a poorer outcome [32].

The modulation of capsule expression (early culture lack of capsule) may have been in part a function of organism response to the CNS immune state that we identified to include a robust Th1 response (elevated levels of INF*γ*, TNF*α*, GRO/CXCL1, IL8, and IL12p40) and simultaneous elevation of levels of the anti-inflammatory cytokine IL10. Low levels of IL6 may also have played a role in persistence of CNS infection in this case. Further characterization of cytokines in infections may eventually be useful in diagnosis given the observed limitations of current analytical methods.

The compilation of our patient's history, exam, lab findings, and imaging strongly suggested a fungal CNS infection that warranted repeated investigations. His indolent course made the diagnosis challenging but was consistent with the longer window from symptom onset to diagnosis in immunocompetent individuals compared with HIV-infected counterparts in a Taiwanese cohort [34]. He had subtle risk factors and exposure opportunities. Zoonotic transmission from his pet cockatoo is possible as a report with probable transmission of* C. neoformans* has been described albeit in an immunocompromised individual [35]. Additionally,* C. neoformans* has been isolated from sewage sludge to which he could be exposed while working in a wastewater treatment plant [36]. An active surveillance program found that both HIV-infected patients and control group patients who were active smokers had a higher risk for cryptococcal infection [37], suggesting an increased risk with his 50-pack-year history.

Our patient's treatment was consistent with the standard of care for* C. neoformans* meningoencephalitis with antifungal therapy, serial LPs and CSF diversion for hydrocephalus, and corticosteroid therapy. Although* Cryptococcus* can produce biofilm on ventriculoatrial and ventriculoperitoneal shunts in patients previously infected with* Cryptococcus* [38], it is not contraindicated and has demonstrated improvement in cognitive impairment, gait, and papilledema in some without associated mortality or morbidity [39, 40].

We advocate an emphasis on collecting a large volume of CSF dedicated for culture at every opportunity for a lumbar puncture when a high index of suspicion exists, despite an immunocompetent host and negative antigen assays, as yeast titers are potentially low, capsule expression may be variable, and host immune response may modulate capsule expression, antigen shedding, and virulence. Since this case, our clinical microbiology department has adopted a rapid 1-hour multiplex PCR based panel for screening 14 potential bacteria, viruses, and* Cryptococcus neoformans/gattii* when considering meningitis/encephalitis (BioFire FilmArray Meningitis/Encephalitis Panel®) which may ameliorate difficulties mentioned here with traditional methods noted above.

## Figures and Tables

**Table 1:** Serial lumbar punctures.

	1	2	3	4
Opening pressure, cm H_2_O (mL removed)	17 (?)	13 (26)	13 (32)	16 (31)
Glucose	19	23	34	47
Protein	300	248	153	158
RBC	46, 0	54, 34	180, 13	29, 0
WBCs	235, 188	213, 303	240, 206	67, 175
% lymphocytes	60, 76	90, 88	93, 95	X, 89
Cryptoantigen	**−**	**−**	**−**	**−**
Fungal culture	**−**	/	/	**+**

1: performed during initial admission, 2: performed 8 mo later, 3: performed 2 days later, and 4: performed 6 days later. “?”: volume of CSF removed during first lumbar puncture was not documented. “X”: cell count for tube 1 not ordered. Pairs of data represent cell count/differential for separate tubes (typically tubes 1 and 4). Reactive (+) and nonreactive (−) cryptococcal antigen assays; fungal culture growth denoted by (+) or absence of growth (−); “/” indicates not performed.
